# Editorial: Metabolic Adjustments and Gene Expression Reprogramming for Symbiotic Nitrogen Fixation in Legume Nodules

**DOI:** 10.3389/fpls.2019.00898

**Published:** 2019-07-09

**Authors:** Brett James Ferguson, Kiwamu Minamisawa, Nacira Belen Muñoz, Hon-Ming Lam

**Affiliations:** ^1^School of Agriculture and Food Sciences, The University of Queensland, Brisbane, QLD, Australia; ^2^Graduate School of Life Sciences, Tohoku University, Sendai, Japan; ^3^National Institute of Agricultural Technology, Córdoba, Argentina; ^4^Center for Soybean Research of The State Key Laboratory of Agrobiotechnology, School of Life Sciences, The Chinese University of Hong Kong, Shatin, Hong Kong

**Keywords:** nitrogen fixation, nodules, metabolic adjustment, gene expression reprogramming, long-range signals, legume, rhizobia

Legumes are important sources of food, feed, and biofuel. Their ability to enter into a symbiotic relationship with nitrogen-fixing rhizobia has a strong impact on environment and agriculture. Maximizing the use of legumes and improving the nodulation and nitrogen fixation processes are seen as pivotal steps toward enhancing agricultural sustainability, where nitrogen fixation has a key role in soil functions, nutrient cycling, soil biodiversity, ecosystem services, and even food security (Foyer et al., 2016; El Mujtar et al., 2019).

The symbiosis involves compatible legume-rhizobia species to recognize one another in the rhizosphere, followed by the bacteria infecting the host and initiating the formation of nodules that would eventually house the bacteria (Ferguson et al., 2010). The environment inside the nodule is ideal for rhizobia to convert atmospheric di-nitrogen gas into alternative nitrogenous compounds that can be used by the host plant. The number of nodules is tightly controlled by the host, based on the balance between its need to acquire nitrogen and its ability to expend resources and energy forming and maintaining these resource-demanding structures (Ferguson et al., 2019). The interaction between legumes and rhizobia is a typical example of co-evolution. For example, in soybean, improvement in nitrogen fixation capacity could be part of the result of the domestication process (Muñoz et al., 2016), whereas adaptive transcription profiles were observed when rhizobia were inoculated into different soybean accessions (Jiao et al., 2018).

Xu et al. demonstrated that humic acids could regulate the quorum sensing capacity of *Sinorhizobium meliloti*, suggesting that water-soluble humic materials could potentially be used to improve rhizobial growth and their symbiotic nitrogen fixation efficiency. This was partially demonstrated using water-soluble humic materials to repress the expression of the quorum sensing gene *expR* by augmenting its interaction with the novel regulator *QsrR*. Hara et al. identified functional nitrogen-fixing bacteria associated with the non-legume *Sorghum bicolor*, showing that the major functional nitrogen-fixing bacteria associating with sorghum roots are unique *Bradyrhizobium* species that resemble photosynthetic and non-nodulating *Bradyrhizobium* strains. Based on these findings, they discussed the nitrogen-fixing potential of the rhizobia-sorghum interaction, and investigated the overall *Bradyrhizobium* diversity. Nguyen et al. worked with *Bradyrhizobium* species and demonstrated that the *innB* gene encodes a novel type III effector that controls symbiosis with *Vigna radiata* (mung bean). Expression of *innB* was shown to be dependent on plant flavonoids and the transcriptional regulator, TtsI. Other effector proteins like innB in the type III secretion system could positively or negatively influence the nodulation and/or nitrogen fixation processes during the co-evolution of rhizobial mutualists with host legume genotypes (Okazaki et al., 2013; Sugawara et al., 2018).

A number of approaches using molecular and functional characterization techniques were used to investigate the roles of various legume signals in nodule development. Rípodas et al. investigated transcription factors of the Nuclear Factor Y (NF-Y) family in *Phaseolus vulgaris*, indicating that both PvNF-YA1 and PvNF-YB7 are part of a network enabling *P. vulgaris* plants to discriminate and select for bacterial strains that perform better at nodule formation, possibly by forming a heterotrimeric complex with PvNF-YC1. This could have wide-ranging implications in the selection of superior varieties for legume nodulation. Sogawa et al. studied the role of SNARE proteins that mediate membrane trafficking in eukaryotic cells, showing that both LjVAMP72a and LjVAMP72b facilitate the legume-rhizobia symbioses and root hair development by affecting the secretory pathway in the host plant. Fernandez-Göbel et al. characterized the rapid systemic redox changes induced during the interactions between soybean and *Bradyrhizobium japonicum*. In addition to molecular genetic studies, they used non-nodulating and super-nodulating soybean mutants to establish that early systemic redox signaling during nodule development depends on Nod factor perception and the induced tolerance response depends on the autoregulation of the nodulation mechanism. In addition, the mini review by Libault highlights current advances in legume nodulation at the level of single cell-types, providing perspectives on the single cell and single cell-type approaches when applied to legume nodulation and how they can be used to enhance the understanding of the complex signaling interactions during rhizobia infection of legume root cells.

The metabolic pathways of carbon, nitrogen, and phosphorus are tightly regulated by the host plant during nodulation and nitrogen fixation. Liu et al. reviewed how host plants balance their needs for these critical resources with those of their microsymbiont partners. They highlighted the network of transporters that traffic these essential metabolites between the two partners through the symbiosome membrane, and how they are regulated by transcription factors that control the expressions of these transporters under different environmental conditions. Conversely, nitrate has been reported to rapidly and reversibly inhibit nodule growth and nitrogen fixation of soybean. Yamashita et al. compared the effects of nitrate to those of ammonium, urea, and glutamine on nodule growth and nitrogen fixation. Interestingly, the long-term supply of nitrate, urea, or glutamine promoted the growth of lateral roots and leaves, which were suppressed by ammonium.

Wongdee et al. and Yang et al. looked at improving nitrogen fixation from different angles. Wongdee et al. investigated *Bradyrhizobium* sp. DOA9, which has the unusual ability to fix nitrogen during both free-living and symbiotic growth. Their findings enhance the understanding of the complex mechanisms that regulate the nitrogenase genes in the DOA9 strain, including the key genes for enabling nitrogen fixation. Yang et al. on the other hand, examined the genetic mechanisms underlying nitrogen fixation in soybean. These authors identified two quantitative trait loci (QTLs) that might be valuable markers for breeding soybean varieties having high biological nitrogen fixation.

This Research Topic aims to highlight current findings in the areas of nodule development and nitrogen fixation, with a focus on the regulation of the molecular factors involved in these processes. The contributing articles are reflections of the highly active research community and the breadth of study in these areas. Collectively, they provide a detailed understanding of the molecular components involved in these processes and point to future directions in the field of legume-rhizobia research.

## Author Contributions

H-ML coordinated the preparation and completed the final version of this editorial. BF wrote the first draft. KM and NM contributed to the writing.

### Conflict of Interest Statement

The authors declare that the research was conducted in the absence of any commercial or financial relationships that could be construed as a potential conflict of interest.

## References

[B1] El Mujtar V. Muñoz N. Prack Mc Cormick B. Pulleman M. Tittonell P. (2019). Role and management of soil biodiversity for food security and nutrition; where do we stand? Glob. Food Sec. 20, 132–144. 10.1016/j.gfs.2019.01.007

[B2] Ferguson B. J. Indrasumunar A. Hayashi S. Lin M.-H. Lin Y.-H. Reid D. E.. (2010). Molecular analysis of legume nodule development and autoregulation. J. Integr. Plant Biol. 52, 61–76. 10.1111/j.1744-7909.2010.00899.x 20074141

[B3] Ferguson B. J. Mens C. Hastwell A. H. Zhang M. Su H. Jones C. H.. (2019). Legume nodulation: The host controls the party. Plant Cell Environ. 42:41–51. 10.1111/pce.13348 29808564

[B4] Foyer C. H. Lam H.-M. Nguyen H. T. Siddique K. H. Varshney R. K. Colmer T. D.. (2016). Neglecting legumes has compromised human health and sustainable food production. Nat. Plants 2:16112. 10.1038/NPLANTS.2016.112 28221372

[B5] Jiao J. Ni M. Zhang B. Zhang Z. Young J. P. W. Chan T.-F.. (2018). Coordinated regulation of core and accessory genes in the multipartite genome of *Sinorhizobium fredii*. PLoS Genet. 14:e1007428. 10.1371/journal.pgen.1007428 29795552 PMC5991415

[B6] Muñoz N. Qi X. Li M.-W. Xie M. Gao Y. Cheung M.-Y.. (2016). Improvement in nitrogen fixation capacity could be part of the domestication process in soybean. Heredity 117, 84–93. 10.1038/hdy.2016.27 27118154 PMC4949726

[B7] Okazaki S. Kaneko T. Sato S. Saeki K. (2013). Hijacking of leguminous nodulation signaling by the rhizobial type III secretion system. Proc. Natl. Acad. Sci. U.S.A. 110, 17131–17136. 10.1073/pnas.1302360110 24082124 PMC3801068

[B8] Sugawara M. Takahashi S. Umehara Y. Iwano H. Tsurumaru H. Odake H.. (2018). Variation in bradyrhizobial NopP effector determines symbiotic incompatibility with *Rj2*-soybeans via effector-triggered immunity. Nat. Commun. 9:3139. 10.1038/s41467-018-05663-x 30087346 PMC6081438

